# 726. Operational Impact of Infectious Disease Threats During Military Travel

**DOI:** 10.1093/ofid/ofab466.923

**Published:** 2021-12-04

**Authors:** Michael A Boatwright, Gregory Utz, Anjali Kunz, Rhonda E Colombo, Jamie Fraser, Huai-Ching Kuo, David Tribble, Tahaniyat Lalani

**Affiliations:** 1 Walter Reed National Military Medical Center, Silver Spring, MD; 2 Uniformed Services University of the Health Sciences, Bethesda, MD; 3 Madigan Army Medical Center, Tacoma, WA; 4 Infectious Disease Clinical Research Program, Bethesda, MD; 5 Henry M. Jackson Foundation for the Advancement of Military Medicine, Inc., Bethesda, MD; 6 Infectious Disease Clinical Research Program/Uniformed Services University, Bethesda, MD; 7 IDCRP, Bethesda, MD; 8 Uniformed Services University, Bethesda, MD; 9 IDCRP, HJF, and NMCP, Bethesda, MD

## Abstract

**Background:**

We evaluated the incidence and operational impact of travelers’ diarrhea (TD), influenza-like illness (ILI) and undifferentiated febrile illness (FI) in US active duty (AD) personnel traveling outside the continental U.S for deployment (DEP), joint military training exercises (EXR) or other military travel (e.g. Temporary Duty Travel) (TDY).

**Methods:**

AD personnel traveling for ≤ 6.5 months were prospectively enrolled between 2010-2019. Participants completed a post-travel survey regarding risk behaviors, illnesses and impact on daily activities. Trip purpose was categorized into DEP, EXR, TDY and syndromic definitions were used to identify cases of TD, ILI and FI based on symptoms. A multivariate logistic regression model with backward selection was used to determine the odds ratio associated with partial or complete incapacitation due to infections (a composite endpoint of either TD, ILI or FI).

**Results:**

1822 servicemembers were enrolled: 36.2% traveled on DEP, 36.2% for EXR and 27.7% for TDY (Table 1). 83.5% of personnel traveling for DEP were Special Operations and Marine units, and 82% of the EXR group participated in Pacific Pathways. Overall, 19% of US personnel experienced infections associated with partial or complete incapacitation (median duration of incapacitation- TD: 1 day; ILI: 4 days; FI: 3 days). DEP personnel had a longer travel duration and the highest rate of partial or complete incapacitation due to TD, ILI or FI (Figure 1 and 2). Risk factors associated with partial or complete incapacitation due to infections were non-adherence with malaria chemoprophylaxis (OR: 1.7 [95%CI:1.2-2.4]), close contact with locals (OR:1.7 [95%CI:1.3-2.2]), inability to clean hands regularly before meals (OR: 1.7 [95%CI: 1.3-2.3]), fresh water or rodent exposure OR: 1.4 (95%CI:1.1-1.9) and consuming street vendor food (OR:1.8 [95%CI:1.3-2.4]).

Table 1. Demographic and travel characteristics of AD personnel traveling outside the continental US.

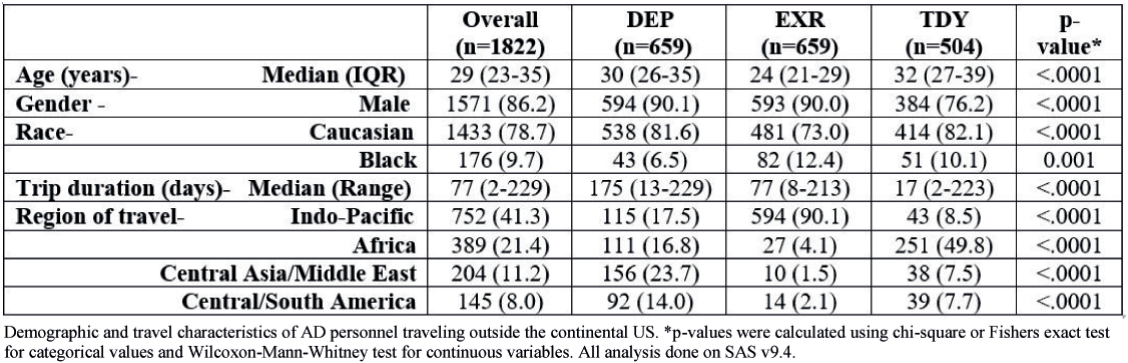

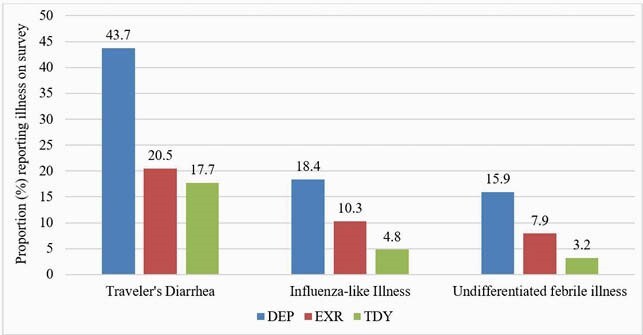

Proportion of AD servicemembers that experienced TD, ILI or undifferentiated febrile illness during DEP, EXR, TDY (p<0.05 for the comparison of each illness between DEP, EXR and TDY).

Figure 2.

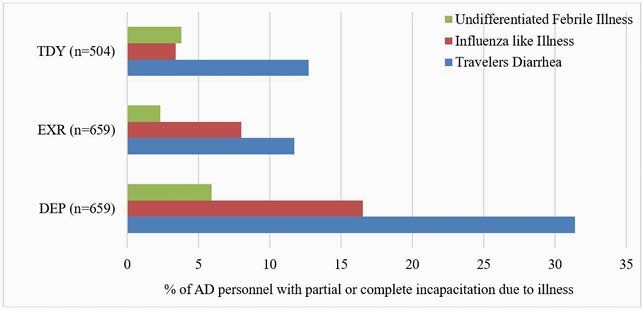

Proportion of AD personnel with partial or complete incapacitation due to TD, ILI or FI during DEP, EXR or TDY (p < 0.05 for the comparison of each illness between DEP, EXR and TDY).

**Conclusion:**

Infectious disease syndromes are common during overseas military travel. TD had the highest negative impact on military travel especially among DEP personnel. We identified several modifiable risk factors associated with incapacitating infections which can be used to inform preventive and treatment strategies.

**Disclosures:**

**All Authors**: No reported disclosures

